# Myocardium-targeted transplantation of PHD2 shRNA-modified bone mesenchymal stem cells through ultrasound-targeted microbubble destruction protects the heart from acute myocardial infarction

**DOI:** 10.7150/thno.43233

**Published:** 2020-04-06

**Authors:** Zhenxing Sun, Yuji Xie, Robert J. Lee, Yihan Chen, Qiaofeng Jin, Qing Lv, Jing Wang, Yali Yang, Yuman Li, Yu Cai, Rui Wang, Zhengyang Han, Li Zhang, Mingxing Xie

**Affiliations:** 1 Department of Ultrasound, Union Hospital, Tongji Medical College, Huazhong University of Science and Technology, Wuhan 430022, China.; 2 Hubei Province Key Laboratory of Molecular Imaging, Wuhan 430022, China; 3 College of Pharmacy, The Ohio State University, Columbus, OH 43210, USA

**Keywords:** UTMD, bone marrow stem cell, PHD2 shRNA, acute myocardial infarction

## Abstract

Ultrasound-targeted microbubble destruction (UTMD) is a promising approach to facilitate the precise delivery of bone marrow stem cells (BMSCs) to the ischemic myocardium. However, stem cell therapy for ischemic myocardium is challenging due to the poor survival of transplanted stem cells under severe ischemic conditions. In this study, we investigated whether myocardium-targeted transplantation of prolyl hydroxylase domain protein 2 (PHD2) shRNA-modified BMSCs by UTMD increases the viability of grafted cells, and enhances their cardioprotective effects in acute myocardial infarction.

**Methods:** BMSCs were transduced with lentiviral PHD2 shRNA, and a novel microbubble formulation was prepared by a thin-film hydration method. In rats, BMSCs with or without PHD2 shRNA modification were transplanted by UTMD after inducing acute myocardium infarction. Effects of PHD2 shRNA on BMSC survival, myocardial apoptosis, angiogenesis, and cardiac function were evaluated. *In vitro*, anti-apoptotic effects and its mechanisms of PHD2 silencing on BMSC and BMSC-conditioned medium on H9C2 cell were detected.

**Results:** PHD2 shRNA-modified BMSC transplantation by UTMD resulted in increased BMSC survival, reduced myocardial apoptosis, reduced infarct size, increased vascular density, and improved cardiac function compared to the control vector-modified BMSC transplantation by UTMD. PHD2 silencing increased BMSC survival through a HIF-1α-dependent mechanism. The decrease in cardiomyocyte apoptosis by conditioned medium from PHD2 shRNA-treated BMSCs was due to an increase in the expression of insulin-like growth factor (IGF)-1.

**Conclusions:** The delivery of PHD2 shRNA-modified BMSCs by UTMD promoted grafted cell homing and activity, and increased myocardial angiogenesis in the infarcted heart, leading to improved cardiac function. This combination may provide a promising strategy for enhancing the effectiveness of stem cell therapy after acute myocardial infarction.

## Introduction

Acute myocardial infarction (AMI), causing loss of cardiomyocytes and scar formation that cannot be reversed, is one of the underlying causes of mortality and morbidity worldwide 1. Transplantation of bone marrow mesenchymal stem cells (BMSC) represents a feasible method to repair ischemic myocardium by releasing paracrine factors into the surrounding tissue, which reverses cardiac remodeling, attenuates cardiac fibrosis, and improves cardiac functions 2, 3. However, intravenously injected stem cells reach ischemic myocardial tissues inefficiently, limiting the effective implementation of BMSC therapy 4, 5. Therefore, the improvement of stem cell homing is a constant challenge in the field of stem cell transplantation.

It has been shown that ultrasound-mediated microbubble destruction (UTMD) can create pores in the capillary walls and alter the local myocardial microenvironment, which promotes engrafted stem cell homing from the blood vessels to the ischemic myocardial tissues 6, 7. Besides, UTMD combined with stem cell transplantation could promote stem cell homing to the infarcted region without negatively affecting the proliferation and survival of the transplanted stem cells 8.

However, another limitation of stem cell therapy to treat ischemic myocardium is the poor survival of transplanted stem cells in the harsh ischemic environment. It has been well documented that the survival of transplanted stem cells is affected by the local environment (e.g., inflammatory cytokines originating from hearts with myocardial infarction) 9, 10. Addressing this challenge is important for improving the curative effect of BMSCs on AMI. One strategy is to increase transplanted cell viability through genetic modification. Hypoxia-inducible factor-1α (HIF-1α) is a vital regulator in adaptation to hypoxic stress. It regulates the response of a variety of genes participating in systemic and cellular responses to hypoxia 11, 12. However, oxygen-dependent prolyl hydroxylase-2 (PHD2) degrades HIF-1α under normoxia 13, 14. Thus, inhibiting HIF-1α degradation by silencing PHD2 is a promising strategy to improve the survival of stem cells.

**Figure 1:**
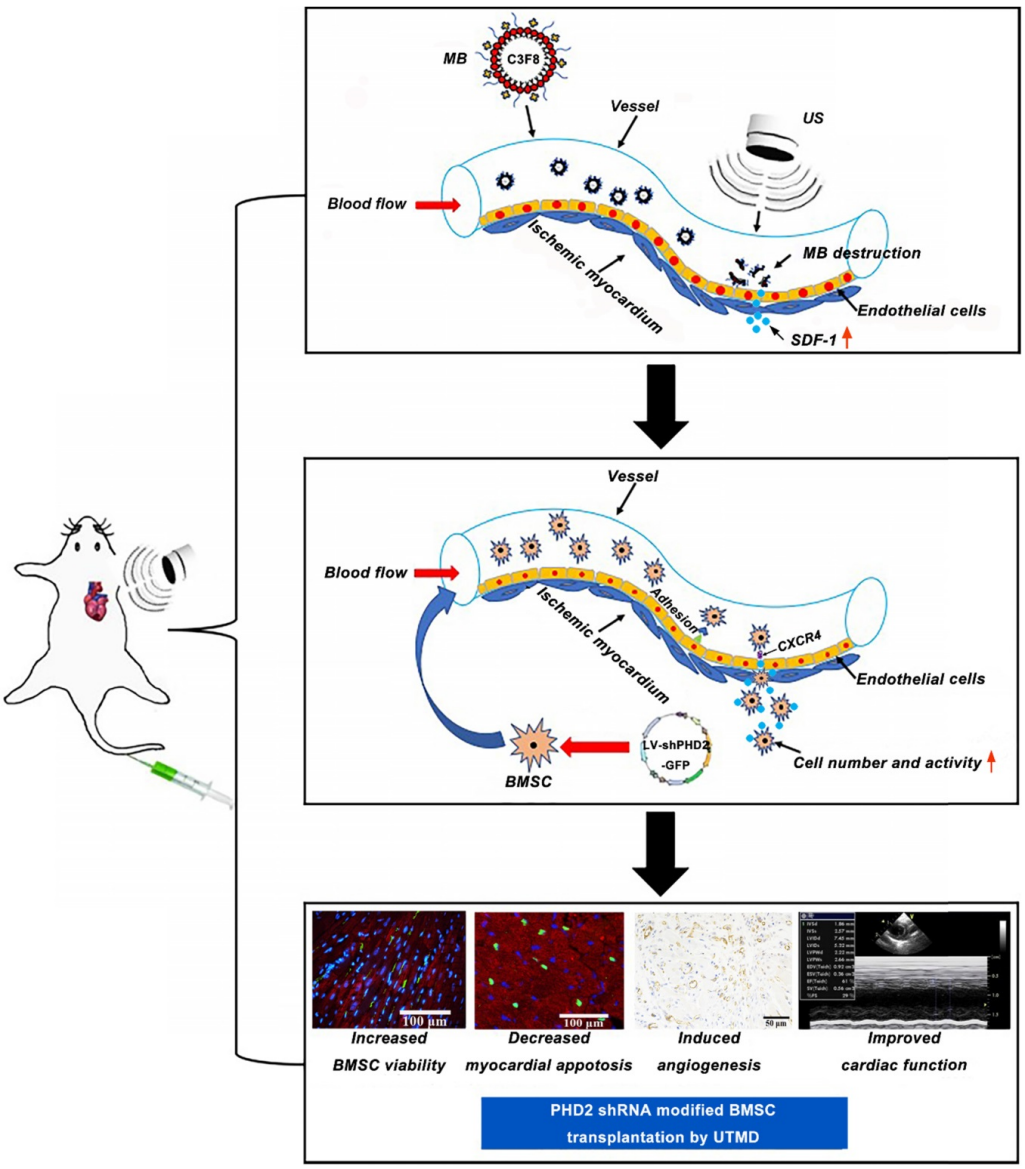
Schematic representation of PHD2 shRNA-modified BMSC transplantation using UTMD for infarcted myocardium repair.

In this study, we hypothesized that myocardium-targeted transplantation of PHD2 shRNA-modified BMSCs by UTMD could increase the therapeutic effect of grafted cells in a rat model with MI. We investigated whether transplantation of PHD2 shRNA-modified BMSCs by UTMD improves cardiac function in myocardial infarction by reversing the myocardial remodeling and reducing the size of infarction in rats. This minimally invasive stem cell transplantation strategy combined with PHD2 shRNA modification could have significant potential for clinical application in patients with AMI. The schematic representation of the UTMD mediated BMSC therapy strategy is shown in Figure 1.

## Methods

### *In vitro* studies

#### BMSC preparation and identification

BMSCs from Sprague Dawley (SD) rats were isolated, as described previously 15. In brief, the anesthetized SD rats (1% pentobarbital sodium, 40mg/kg) were executed, and bone marrow of femurs and tibias were cleaned and flushed by PBS. Based on density gradient centrifugation, mononuclear cells were separated before being incubated in low-glucose Dulbecco's modified Eagle's medium (DMEM; Gibco, Grand Island, NY, USA) containing streptomycin (100g/mL), penicillin (100 U/mL), and 10% fetal bovine serum (FBS; Gibco). Nonadherent cells were removed after 48 h. The passaged cells were cultured in a humidified incubator with 5% CO_2_ at 37 °C. The cell-culture medium was changed every two days. Once the BMSCs reached 80% confluence, the primary cultures were passed to 2 new flasks. The morphology and typical surface markers were ascertained by flow cytometry to identify the BMSCs as described in previous studies 16, 17.

#### Lentivirus preparation and transduction into BMSCs

Shanghai Genechem Co. Ltd., Shanghai, China, provided short hairpin RNA (shRNA), lentiviral transduction particles against PHD2 (LV-shPHD2-GFP), and control non-target shRNA (LV-GFP). All lentiviral vectors contained the green fluorescent protein (GFP) reporter.

Third-generation BMSCs were seeded in 24-well plates. Cells were grown to 80% confluence and then split into three experimental groups: Control group (non-transfected), LV-GFP group (transfected with LV-GFP), and LV-shPHD2-GFP group (transfected with LV-shPHD2-GFP). BMSCs were transfected with lentivirus at a multiplicity of infection (MOI) of 20 in the presence of 8μg/ml Polybrene following the manufacturer's instructions (Santa Cruz Biotechnology, Dallas, TX, USA).

The infection efficiency was ascertained by the assessment of the GFP expression level by fluorescence microscopy. The transfected BMSCs were sub-cultured for three days in puromycin-containing growth medium and analyzed for the expression levels of PHD2 and HIF-1α by Western blotting and real-time PCR (RT-PCR). The sequence of the primers and primary antibodies used are presented in Table 1 and Table 2, respectively. All experiments were performed in triplicate.

#### Analysis of angiogenesis factors after PHD2 gene silencing

Third generation BMSCs were plated in 24-well culture plates and split into three experimental groups: Control (non-transfected), LV-GFP, (transfected with LV-GFP), and LV-shPHD2-GFP (transfected with LV-shPHD2-GFP). Supernatants were harvested 72 h after transfection. The concentrations of basic fibroblast growth factor (bFGF) and vascular endothelial growth factor (VEGF) in the supernatant were determined with a Quantikine ELISA kit (R&D Systems, Minneapolis, MN, USA). The experiment was conducted 3 times separately.

#### Effects of PHD2 silencing on BMSC resistance to oxygen-glucose deprivation injury

To examine the cell protective effect of PHD2 silencing in BMSCs, an *ex-vivo* model of oxygen-glucose deprivation (OGD) was established. Third generation BMSCs were plated in 24-well culture plates and split into four groups: (A) Control-BMSC (non-transfected), (B) BMSC (non-transfected), (C) LV-GFP-BMSC (transfected with LV-GFP), and (D) LV-shPHD2-GFP-BMSC (transfected with LV-shPHD2-GFP with or without LV-shHIF-1α) 24 h after inoculation. BMSCs in the latter three groups [(B), (C) and (D)] were cultured in glucose-free DMEM and then exposed to hypoxic conditions (5% CO2 and 95% N2) for 6h. Cell apoptosis was demonstrated with flow cytometry. The effect of PHD2 on expression levels of activated caspase-3 and total caspase-3 and downstream genes (HIF-1α, EPO) involved in shPHD2-mediated cell protection against OGD injury were assessed by Western blotting. The primary antibodies used are listed in Table 2. The experiments were performed in triplicate.

#### Anti-apoptotic effect of BMSC-conditioned medium (CM) on H9C2 cells

CM from BMSCs in BMSC, LV-GFP-BMSC, and LV-shPHD2-GFP-BMSC groups was used to treat H9C2 cells in 24-well plates. H9C2 cells were split into five groups and treated with Control (untreated with OGD), OGD, BMSC-CM, LV-GFP-BMSC-CM, and LV-shPHD2-GFP-BMSC-CM, respectively. After 24h of culture at 37°C and 5% CO_2_, H9C2 cells were subjected to OGD for 6 h. The cell apoptosis was analyzed by an apoptosis kit (Multi Sciences Biotech Co., Ltd.) according to the manufacturer's instructions (Multi Sciences Biotech Co., Ltd.). Cellular levels of activated and total caspase-3 were detected by Western blot analysis. Furthermore, the expression of the anti-apoptosis gene (IGF-1) in the CM from LV-shPHD2-GFP-BMSC-mediated myocardial cell protection against OGD injury was assessed by ELISA (Abcam, Cambridge, MA, USA). The primary antibodies used are listed in Table 2. The expression levels in all experiments were measured in triplicate.

#### Preparation of the microbubbles (MBs)

MBs consisting of a coating of 3-[N-(N',N'-dimethylaminoethane)-carbamoyl] cholesterol (DC-CHOL), 1,2-distearoyl-sn-glycerol-3-phosphoethanolamine-N-[maleimide (polyethylene glycol)] (DSPE-PEG2000), and dipalmitoyl phosphatidylcholine (DPPC) with a octafluoropropane (C3F8) gas core (F2 Chemicals, Preson, UK) were constructed by the thin-film hydration method as described in our previous study 18. The concentration of MBs was regulated to 1×10^9^ MBs/ml by diluting the MBs solution with PBS. Subsequently, the MB solution was disinfected by ^60^Co-γ radiation and kept at 4°C for subsequent applications.

#### Characterization of the MBs

The morphology and size distribution of the MBs were visualized with an optical microscope (model IX70, Olympus Inc., Melville, NY, USA) and a transmission electron microscope (TEM, Hitachi H-7000FA, Japan). The zeta potential, concentration, and size distribution of the MBs were determined with the use of a Zetasizer NANO ZS system (Malvern Instruments Ltd., Malvern, UK). The concentration and diameter of MBs at various time points were measured, and the imaging ability of the MBs at different concentrations was assessed with a homemade agarose phantom *in vitro*. Also, the imaging properties of the MBs for rat hearts were evaluated *in vivo*. The images were acquired and analyzed using a clinical IU22 ultrasound scanner (Philips Medical Systems, Amsterdam, Netherlands).

### *In vivo* studies

The Animal Breeding and Research Center of Tongji Medical College, Huazhong University of Science and Technology, China provided two-month-old adult male Sprague-Dawley (SD) rats (200∼220 g). The Huazhong University of Science and Technology Animal Care and Use Committee authorized all animal studies. The animal procedures conformed to the guidelines from Directive 2010/63/EU of the European Parliament on the protection of animals used for scientific purposes.

#### Animal surgery to induce AMI

The MI model was established following the existing guidelines. Briefly, all rats were treated with tracheal intubation ventilation by a rodent ventilator (Shanghai Yuyan Instruments, China) following intraperitoneal anesthesia using 1% pentobarbital sodium (40 mg/kg, administered intraperitoneally). The left coronary artery was ligated 2-3 mm from the tip of the left auricle with a 6/0 suture to trigger AMI. Evidence of an AMI was confirmed by the appearance of a Q wave and S-T segment elevation on an electrocardiogram.

#### SDF-1 expression in the AMI area following UTMD

AMI rats were separated into four groups: (i) Control, PBS (0.5 mL) infusion; (ii) MB (0.5 mL) infusion; (iii) Ultrasound, PBS (0.5 mL) infusion; (iv) UTMD, MB (0.5 mL) infusion. During the ultrasound irradiation, a micropump was used to infuse 0.5 mL of MB or PBS at a rate of 15 ml/h.

All AMI rats were executed in each group under anesthesia with 1% pentobarbital sodium (40 mg/kg, administered intraperitoneally) after ultrasonic irradiation. Hearts were perfused with saline solution and rapidly collected. Some rat hearts were immersed in 10% paraffin. The sections (5μm) were dewaxed and stained with the use of an antibody against SDF-1. Images were captured using an inverted microscope (model IX70, Olympus Inc. Melville, NY) after visualization with diaminobenzidine (DAB). Other rats' myocardium from the peri-infarct regions were rapidly frozen in liquid nitrogen. Protein expression levels were analyzed by Western blotting. The primary antibodies used are presented in Table 2. The experiments were performed in triplicate.

#### Effect of UTMD on BMSC migration

To evaluate the improvement of BMSC engraftment mediated by UTMD, all rats were divided into four groups: (i) Sham, PBS (250μL); (ii)MI, PBS (250 μL); (iii) MI+SDF-1, (250 μg plasmid DNA solution in 250 μl of PBS); (iv) UTMD, PBS (250μL). BMSCs (1 ml, 10^6^/ml) were injected through the caudal vein after myocardial injection of PBS or plasmid DNA around the border zone and UTMD treatment. The expression of SDF-1 in the infarct border area at 1, 3, 4, and 7 days was evaluated with a Quantikine ELISA kit (R&D Systems, Minneapolis, MN, USA) according to the manufacturer's protocol. The number of homing cells in ischemic myocardium and other organs was tested by RT-PCR for the Y chromosome‐specific Sry gene 24h after stem cell transplantation as described in a previous study 19, and the ratio of the number of grafted cells in the heart or in other organs to initially injected cells as a percentage.

#### BMSC transplantation for myocardial infarction by UTMD

Forty rats were split into four groups: (i) sham, PBS (1 mL) infusion only; (ii) MI, PBS (1 mL) infusion only; (iii) MI-LV-GFP-BMSC, 1×10^6^ LV-GFP infected cells suspended in 1mL of PBS infusion; (iv) MI-LV-shPHD2-GFP-BMSC, 1×10^6^ LV-shPHD2 infected cells suspended in 1mL of PBS infusion.

Rats in the MI-LV-GFP-BMSC and MI-LV-shPHD2-GFP-BMSC groups underwent UTMD followed by BMSC transplantation. A micropump was used to infuse 0.5 mL of MB at a rate of 15 ml/h during ultrasound irradiation. Rats were ultrasonically treated with a sonoporator at the frequency of 1 MHz, the power of 2.0 W/cm^2^, duty cycle 50%, as well as 1 kHz pulse repetition frequency for two minutes. The ultrasound probe was fixed on the canine cardiac papillary muscle short-axis. Following UTMD, BMSCs (1 mL) were injected through the caudal vein. Rats in the sham and MI groups received UTMD prior to receiving 1 mL PBS injection. BMSC transplantation mediated by UTMD was performed at 2-day intervals between Day 0 and Day 4. A dose of furosemide (0.4 mg/kg) was injected before BMSC transplantation by UTMD to prevent congestive heart failure because of the volume overload.

#### Analysis of the survival of engrafted BMSCs *in vivo*

To confirm that PHD2 shRNA modification could enhance BMSC survival, its grafting efficiency was analyzed. All rats were killed 48h after cell transplantation using 1% pentobarbital sodium (40 mg/kg, administered intraperitoneally) and hearts were obtained quickly. The viability of transplanted cells was assessed by the number of fluorescent stem cells in frozen heart sections (5μm). The number of fluorescent stem cells in each rat from the four groups was assessed by counting five randomly selected fields under a fluorescent microscope (model IX70, Olympus Inc., Melville, NY, USA) and quantitated using Image-Pro Plus version 6.0 software (Media Cybernetics, Bethesda, MD) by two observers blinded to the conditions.

#### Detection of cardiomyocyte apoptosis

The TUNEL method was employed to detect levels of cell apoptosis in the infarct border zone following the manufacturer's protocol (Roche Applied Science, South San Francisco, CA, USA). Apoptotic cardiomyocytes of each sample were assessed in 6 randomly selected fields per section and then quantitated using Image-Pro Plus version 6.0 software (Media Cybernetics, Bethesda, MD) by two blinded observers. The average number of TUNEL positive cells per square millimeter (mm^2^) was calculated to assess myocardial apoptosis.

#### Echocardiographic analysis of the left ventricular function

Transthoracic echocardiography was performed to evaluate left ventricular function before the operation and 4 weeks after stem cell transplantation. The GE Vivid 7 system outfitting a 10 MHz transducer was employed by an investigator blinded to group designation. The left ventricular ejection fraction (LVEF) and left ventricular fractional shortening (LVFS) of rats were obtained by M-Mode echocardiography after intraperitoneal anesthesia with 1% pentobarbital sodium (40 mg/kg). Dimension data are presented as the average of measurements of three cardiac cycles.

#### Measurement of infarction size

After the 4-week treatment, rats underwent anesthesia with 1% pentobarbital sodium (40 mg/kg) and then cardiac perfusion with saline. The hearts were quickly harvested and stained with 2,3,5-triphenyltetrazolium (TTC) and Masson's trichrome staining to quantify the extent of infarct size in the left ventricle. Infarct size was measured as the ratio of the average scar circumferences of the endocardium and epicardium to the average left ventricular circumferences of the endocardium and the epicardium, and estimated using Image-Pro Plus version 6.0 software (Media Cybernetics, Bethesda, MD) by two observers blinded to the experiment.

#### Determination of angiogenesis factors and capillary density *in vivo*

Four weeks after BMSC transplantation by UTMD, MI rats were anesthetized with 1% pentobarbital sodium (40 mg/kg) and killed by excision of the heart. Some rats' myocardium from the peri-infarct regions were immersed in liquid nitrogen. Western blot analysis was used to detect the expression level of VEGF and bFGF in ischemic cardiac tissue. Other rat hearts were embedded in 10% paraffin. Dewaxed sections (5μm) were treated with the primary antibody CD31 listed in Table 2. Images were obtained by an inverted microscope (model IX70, Olympus Inc. Melville, NY) after visualization with diaminobenzidine (DAB). Immunoreactivity for CD31 was calculated using the Image-Pro Plus 6.0 analysis system software (Media Cybernetics, Bethesda, MD). Capillaries were defined by positive staining for CD31. The number of cardiac microvessels was counted as described in previous studies 16, 18.

### Statistical analysis

All results were presented as mean ± SD. The data were analyzed using SPSS 19.0 software. Statistical analyses were either performed with the Student's t-test or by one-way ANOVA. A probability value < 0.05 was considered to be statistically significant.

## Results

### Characterization of cultured BMSCs

The morphological features of the cells were observed under an inverted microscope, and the characteristic surface markers were detected by flow cytometry. Most bone marrow cells were spindle-like mesenchymal stem cells and turned more uniform after a few passages (**Figure S1A**). Flow cytometry confirmed their mesenchymal origin; the hematopoietic stem cell markers CD34 and CD45 were negative, while mesenchymal stem cell markers CD29 and CD90 exhibited high expression levels (**Figure S1B**). Thus, we confirmed that the major population of adherent cells was BMSCs.

### Target gene and protein expression in BMSCs after PHD2 silencing

**Figure 2:**
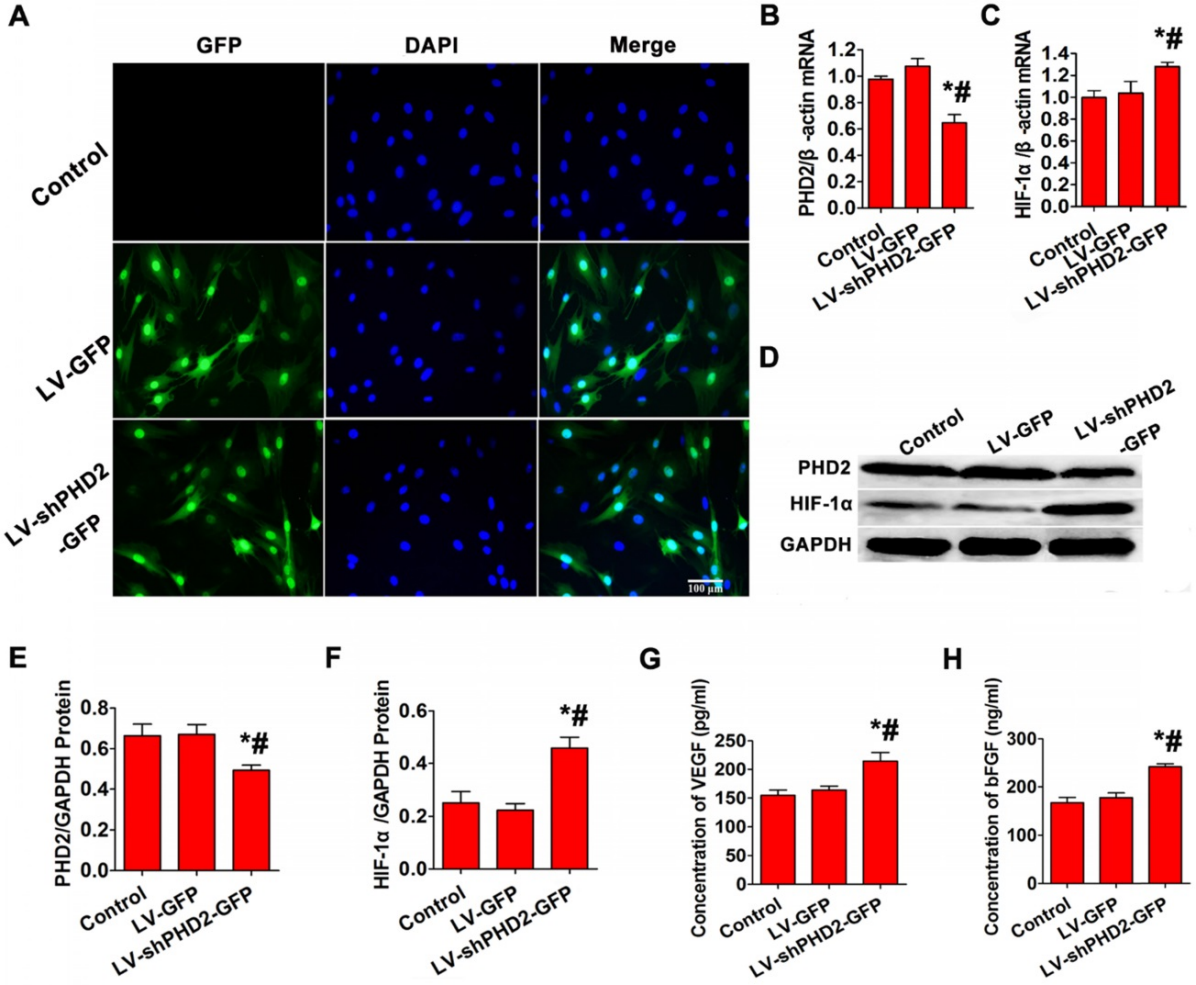
** PHD2, HIF-1α, and angiogenic gene expression analysis after *in vitro* PHD2 gene silencing. (A)** Fluorescence microscopic examination of gene-transferred BMSCs. In the Control group, no GFP-positive cells were detected. More than 90% of GFP-expressing cells were found in the LV-GFP and LV-shPHD2-GFP groups. Bar, 100μm. **(B-C)** RT-PCR showed that PHD2 expression was decreased and HIF-1α expression was up-regulated after BMSCs were transfected with LV-shPHD2-GFP. **(D-F)** Western blotting analysis of PHD2 and HIF-1α expression levels showing down-regulation of PHD2 and up-regulation of HIF-1α after BMSCs were transfected with LV-shPHD2-GFP. **(G)** Concentrations of VEGF in the CMs from different groups. VEGF protein had a higher level in the LV-shPHD2-GFP group as compared to Control and LV-GFP groups. **(H)** Concentrations of bFGF in the CMs from different groups. The level of bFGF protein was higher in the LV-shPHD2-GFP group as compared to Control and LV-GFP groups. Values are mean ± SD. Significant differences were determined by using one-way ANOVA. N = 6/group. * P < 0.017 vs. control, # P < 0.017 vs. LV-GFP.

No fluorescent protein was expressed in the control group, but more than 90% of cells expressed GFP in LV-GFP and LV-shPHD2-GFP groups (**Figure 2A**). RT-PCR indicated that the relative expression level of PHD2 mRNA was significantly lower, and the relative expression level of HIF-1α mRNA was noticeably higher in the LV-shPHD2-GFP group compared with the LV-GFP and control groups (**Figure 2B-C**). Simultaneously, PHD2 protein expression in the LV-shPHD2-GFP group was remarkably lower, and downstream HIF-1α protein expression was significantly higher compared to the other groups (**Figure 2D-F**). These results indicated that LV-shPHD2-GFP could effectively silence the PHD2 gene and activate pathways associated with downstream HIF-1α.

### Increased secretion of angiogenic factors after PHD2 silencing

To study whether the secretion of angiogenic factors was increased after PHD2 silencing, bFGF and VEGF levels in the CM of BMSCs were tested by ELISA. The CM from the LV-shPHD2-GFP-treated cells had noticeably higher levels of VEGF and bFGF than the control and LV-GFP groups (**Figure 2G-H**). Thus, PHD2 silencing was capable of facilitating VEGF and bFGF secretion in BMSCs, which could induce revascularization in ischemic myocardium.

### PHD2 silencing incurs cytoprotection

**Figure 3:**
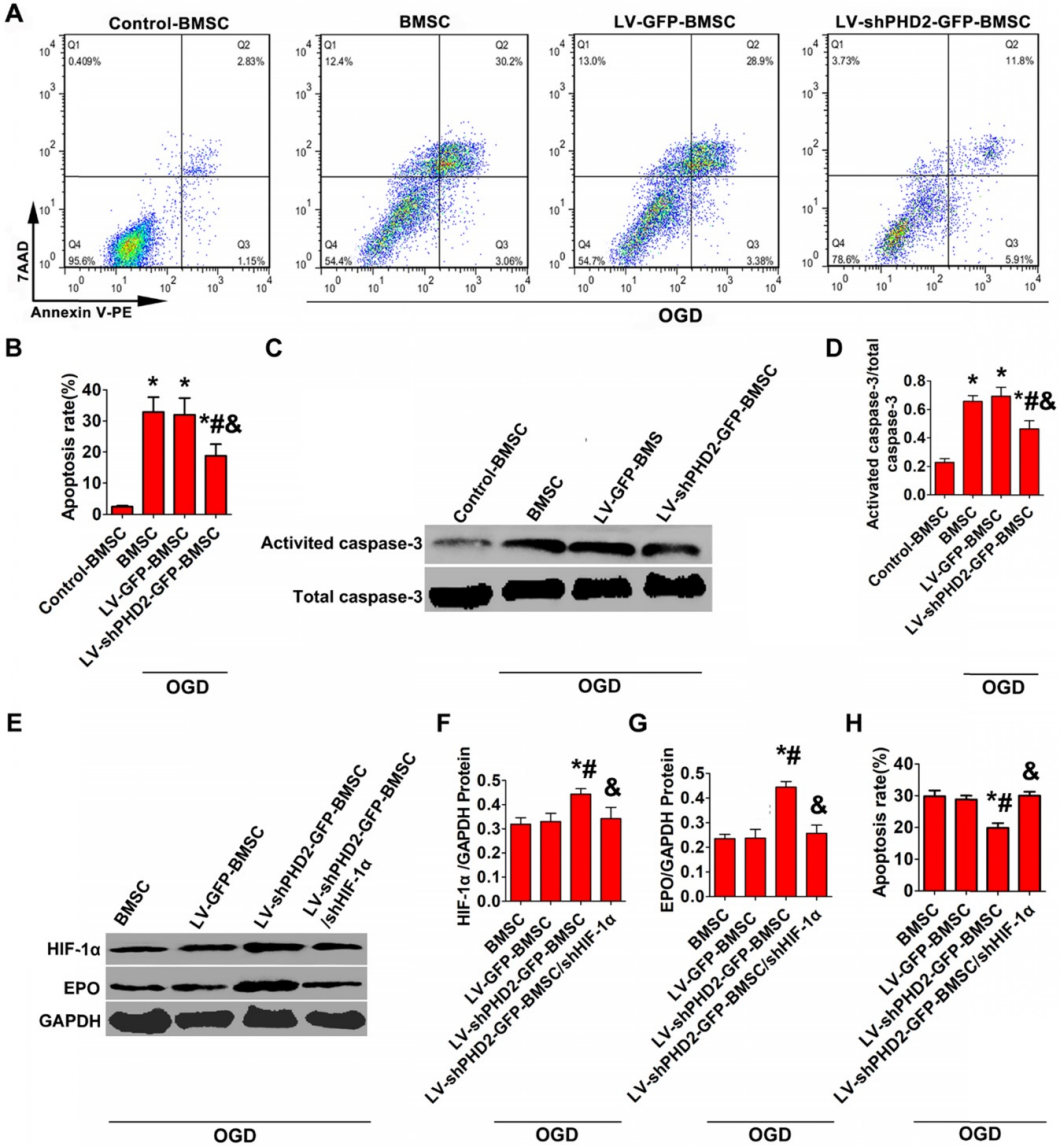
** PHD2 RNA modification reduced BMSCs apoptosis via the HIF-1α-dependent pathway. (A)** Flow cytometry analysis of BMSC apoptosis after Annexin V-PE/7-AAD double staining. BMSCs were transfected with LV-GFP or LV-shPHD2-GFP after the establishment of the oxygen-glucose deprivation (OGD) model *in vitro*. **(B)** Quantitative study of the apoptotic rate of BMSCs. The number of BMSC apoptosis significantly decreased in the LV-shPHD2-GFP group. **(C-D)** Representative blots and quantification of Western blotting analysis of activated caspase-3 expression in BMSCs. The expression of activated caspase-3 significantly decreased in the LV-shPHD2-GFP group. Values are mean ± SD. Significant differences was determined by using one-way ANOVA. N =6/group. * P < 0.0125 vs. Control-BMSC; # P < 0.0125 vs. BMSC; & P < 0.0125 vs. LV-GFP-BMSC. **(E-G)** Western blotting analysis of HIF-1α and EPO expression in BMSCs and the signal intensities of the blots. HIF-1α and EPO expressions in BMSCs transfected with LV-shPHD2-GFP was significantly increased. **(H)** Apoptotic inhibition capability was lost after BMSCs were transfected with LV-shPHD2-GFP/shHIF-1α. The BMSC apoptosis rate was measured by flow cytometry following annexin V-PE/7-AAD double staining. Values are mean ± SD. Significant differences were determined by one-way ANOVA. N = 6/group. * P < 0.0125 vs. BMSC; # P < 0.0125 vs. LV-GFP-BMSC; & P < 0.0125 vs. LV-shPHD2-GFP-BMSC.

Hypoxic/ischemic stress in the peri-infarct regions of the post-MI heart is considered the major reason for the death of transplanted BMSCs and resident cardiomyocytes. We treated BMSCs with OGD to simulate hypoxic/ischemic stress *in vitro*. Western blot analysis of activated caspase-3 and FACS with Annexin V staining were used to evaluate BMSC apoptosis. Treatment with OGD up-regulated the expression of activated caspase-3 and increased the number of Annexin V-PE/7-AAD-positive cells in all groups. However, PHD2 silencing noticeably decelerated the increase in Annexin V-PE/7-AAD-positive cells and activated caspase-3 (**Figure 3A-D**).

The decrease of BMSC apoptosis is thought to be triggered by PHD2 knockdown through activating the HIF-1α signaling pathway. We investigated the mechanism of PHD2 shRNA-mediated cell protection against OGD by analyzing the expression of HIF-1α and its downstream target EPO by Western blotting. The results in **Figure 3E-G** revealed that HIF-1α and EPO protein levels were increased significantly in the LV-shPHD2-GFP-BMSC group compared with other groups in the presence of OGD. However, the anti-apoptotic effect of shPHD2 on BMSCs was lost once HIF-1α was silenced in BMSCs, as shown in **Figure 3H**.

**Figure 4:**
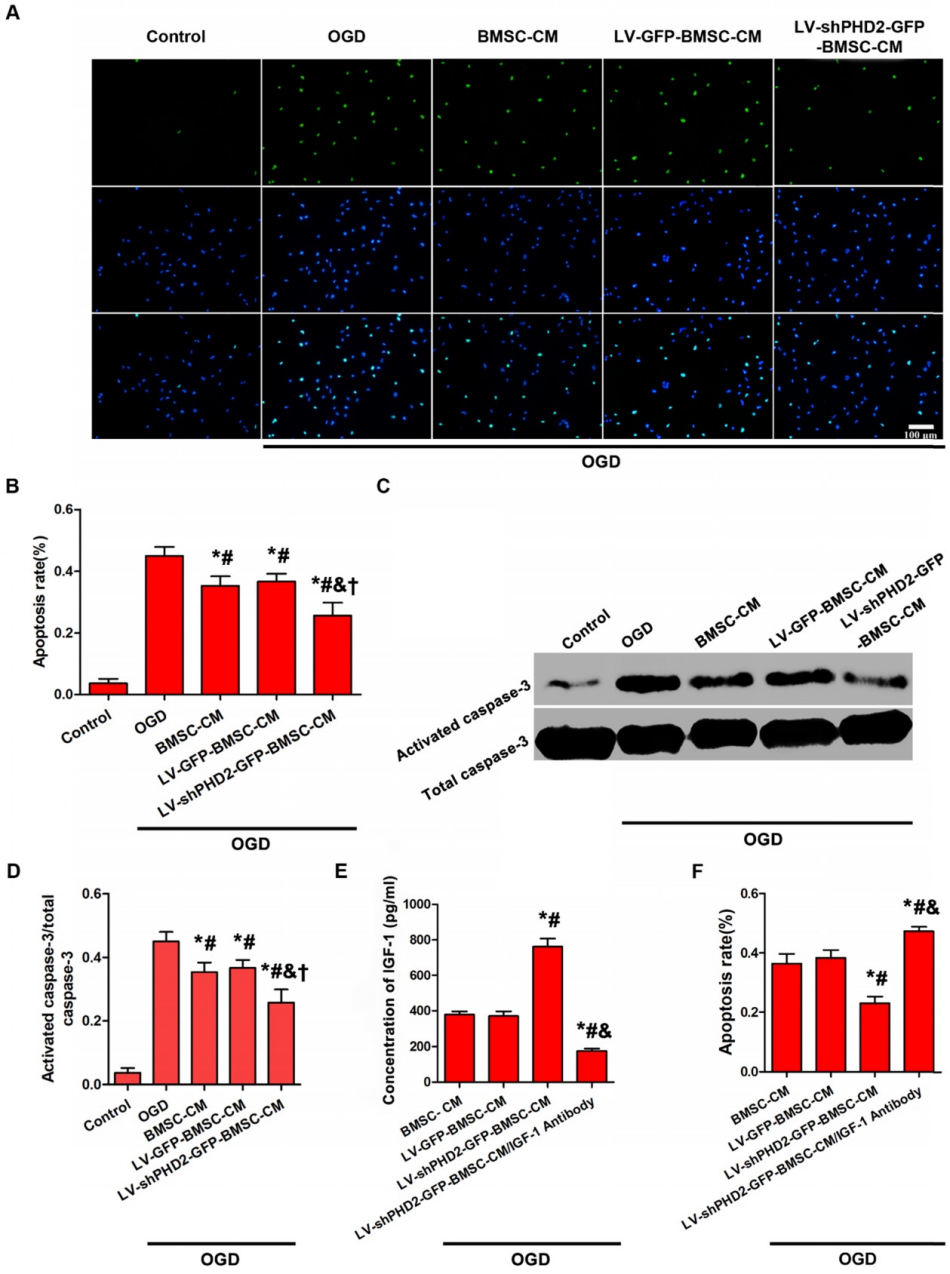
** Anti-apoptotic effect of CM from PHD2-modified BMSC on H9C2 myocardial cells subjected to OGD. (A)** H9C2 cell apoptosis was detected by TUNEL assay in the Control, OGD, BMSC-CM, LV-GFP-BMSC-CM, and LV-shPHD2-GFP-BMSC-CM groups. The cell nuclei were stained with DAPI. TUNEL+ nuclei were labeled with TMR-green. Bar, 100μm. **(B)** Quantitative study of the apoptotic cell ratios by counting the number of positive cells. The fewest number of apoptotic cells in the LV-shPHD2-GFP-BMSC-CM group at 6 hours after OGD treatment in contrast with other groups. TUNEL positive rate= (TUNEL positive nuclei / DAPI positive nuclei) × 100%. **(C-D)** Representative blots and quantification of Western blot analysis of activated caspase-3 of H9C2 cells treated with OGD. The activated caspase-3 of H9C2 subjected to OGD was significantly decreased by CM from LV-shPHD2-GFP-BMSC. Values are mean ± SD. Significant differences were determined by using one-way ANOVA. *p<0.01 vs. Control; # p<0.01 vs. OGD; & p<0.01 vs. BMSC-CM; † p<0.01 vs. LV-GFP-BMSC-CM. **(E)** Level of IGF-1 protein decreased following treatment with CM from LV-shPHD2-GFP-BMSC/IGF-1 antibody. **(F)** Apoptotic inhibition capability was lost after H9C2 cells were treated with CM from LV-shPHD2-GFP-BMSC/IGF-1 antibody. Values are mean ± SD. Statistical significance was determined by using one-way ANOVA. N = 6/group. *p<0.0125 vs. BMSC-CM; # p<0.0125 vs. LV-GFP-BMSC-CM; & p<0.0125 vs. LV-shPHD2-GFP-BMSC-CM.

PHD2 shRNA-modified BMSCs could protect the ischemic myocardium via a paracrine mechanism. The influence exerted by the CM from BMSCs, with or without PHD2 shRNA modification, on OGD-induced H9C2 cell apoptosis was assessed. H9C2 cells were subjected to OGD for 6 h. Compared with the OGD group, the number of TUNEL-positive cells moderately dropped in BMSC-CM and LV-GFP-BMSC-CM groups, but TUNEL-positive cells significantly decreased in the LV-shPHD2-GFP-BMSC-CM group (**Figure 4A-B**). Treatment of H9C2 cells with OGD caused significant activation of caspase-3. BMSC-CM and LV-GFP-BMSC-CM moderately down-regulated the expression of activated caspase-3, but it was significantly down-regulated by LV-shPHD2-GFP-BMSC-CM (**Figure 4C-D**).

The cardioprotective factor secreted from LV-shPHD2-GFP-BMSCs was determined by the ELISA method. The decrease of myocardial apoptosis is hypothesized to be induced by CM from LV-shPHD2-GFP-BMSCs by activating the IGF-1 receptor pathway. ELISA analysis demonstrated that IGF-1 was noticeably up-regulated in the LV-shPHD2-GFP-BMSC-CM group as compared with BMSC-CM and LV-GFP-BMSC-CM groups (**Figure 4E**). However, the anti-apoptotic effect of CM from LV-shPHD2-GFP-BMSCs was substantially decreased when the IGF-1 was neutralized by the IGF-1 antibody (**Figure 4F**).

### Characterization of the MBs

**Figure 5:**
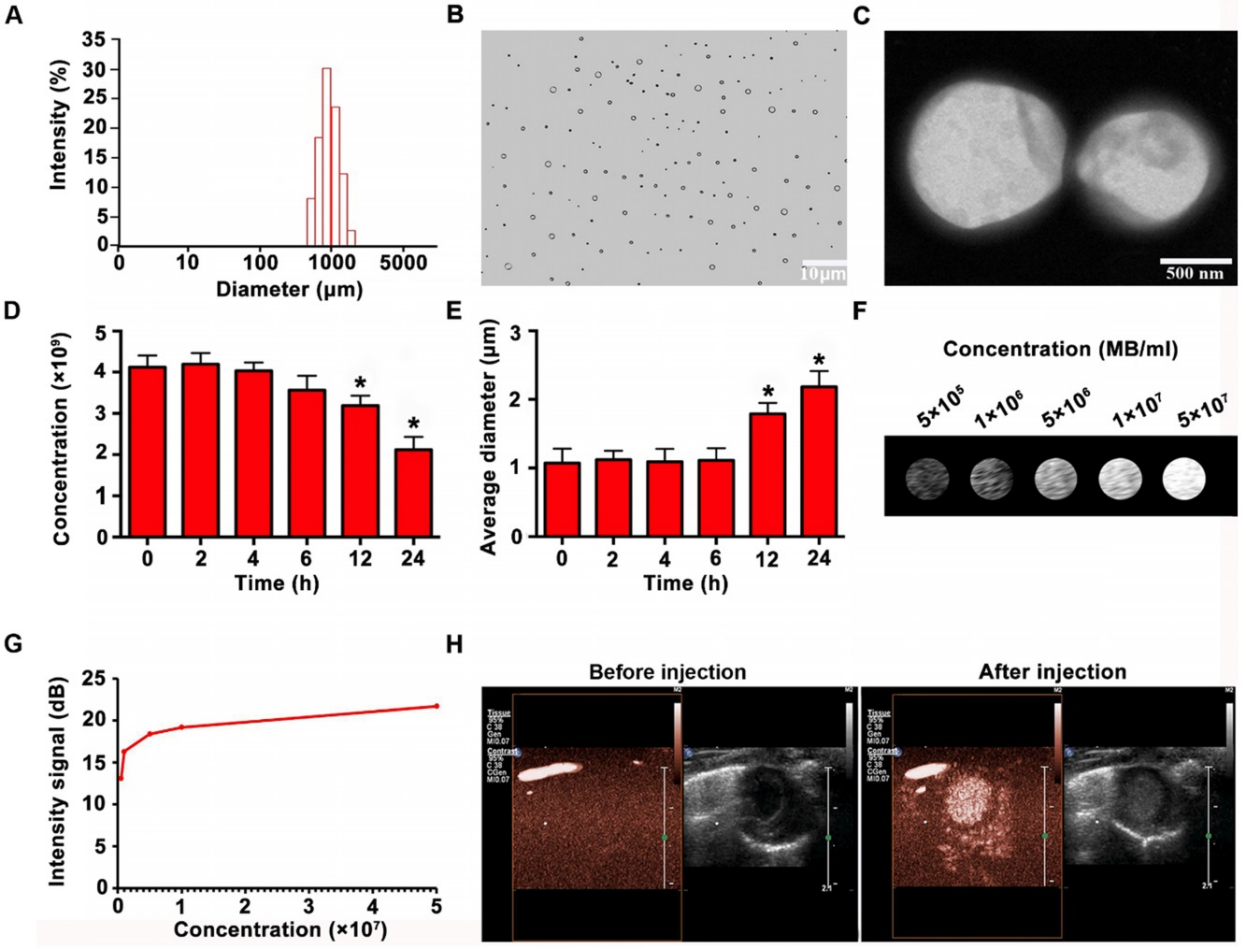
** Characterization and imaging properties of MBs. (A)** Size distribution of MBs **(B-C)** Morphology of MBs observed by optical microscope and by TEM, showing that the MBs were spherized, uniform in size and well distributed. **(D)** Concentration change of MBs at different time points **(E)** Mean diameter change at various time points **(F)** *In vitro* imaging of MBs at different concentrations** (G)** Quantitative analysis showed that the contrast signal increased with increasing MB concentration. **(H)** *In vivo* imaging of MBs for the heart. N = 6/group. Values are mean ± SD. Significant differences were determined by using Student's t-test. * P < 0.05 vs. 0 h.

**Figure 5A** reveals the size distribution of MBs. Optical microscope and electron microscope images showed that the MBs were spherized, well distributed, and even in size (**Figure 5B-C**). The concentration of MBs was 4.12 ± 0.29 ×10^9^/ml. The synthesized MBs had a zeta potential of 27.18 ± 3.32mV and had a mean diameter of 1.07 ± 0.21μm. The MB properties are listed in Table 3. The MBs had prominent stability in concentration and diameter 6 h after preparation. Then MB concentration reduced slightly at 12 h, and significantly at 24h (**Figure. 5D**). Besides, the average diameter of the MBs increased slightly at 12 h and significantly at 24 h (**Figure 5E**). According to imaging results *in vitro*, the signal of MBs enhanced with the concentration increase (**Figure 5F-G**). As shown in **Figure. 5H**, there were no ultrasound imaging signals in the B-mode before injection of MBs, whereas the ultrasound imaging signals were obvious after injection of MBs.

### Increased SDF-1 expression and BMSC migration induced by UTMD

**Figure 6:**
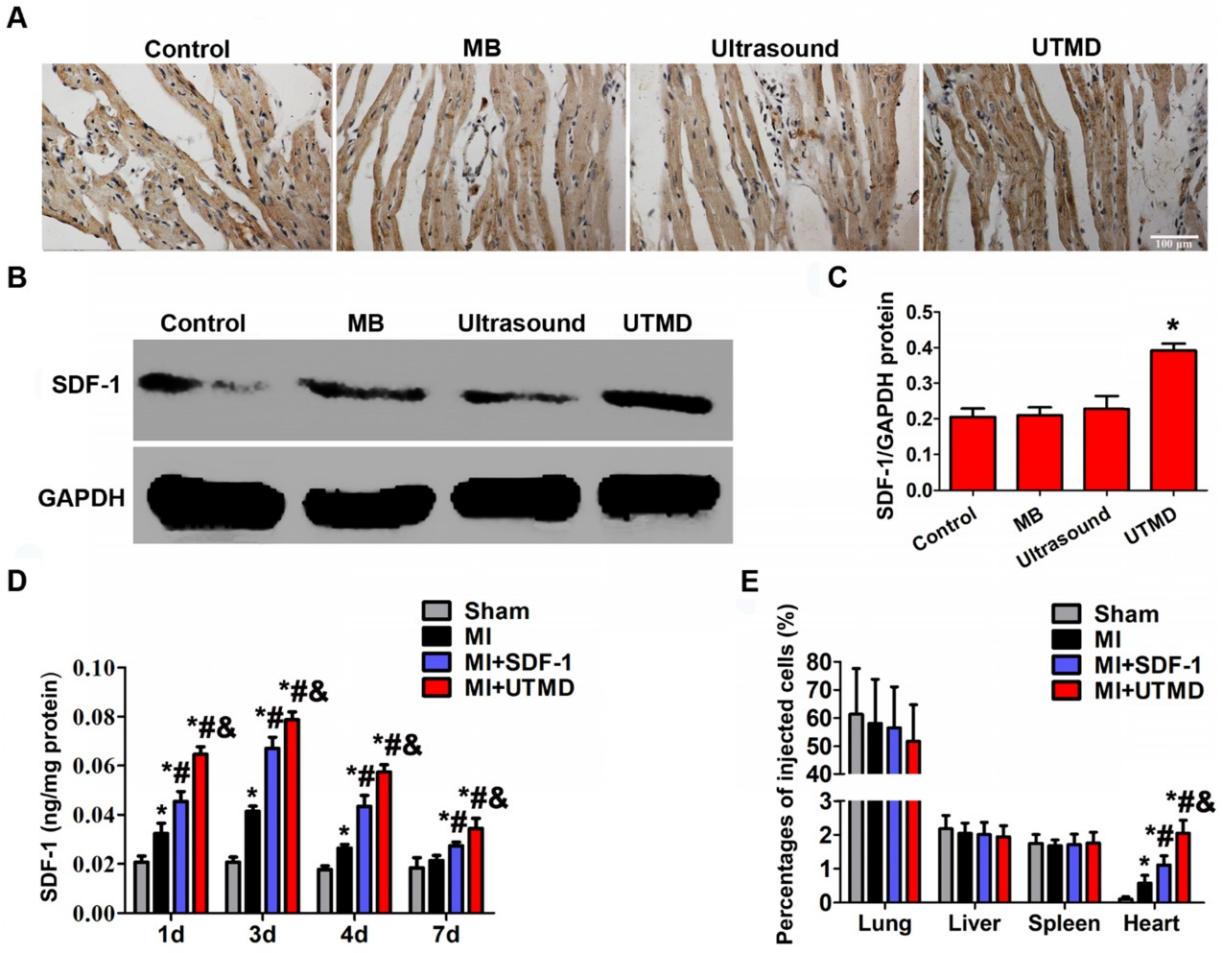
** Expression of SDF-1 and BMSC migration in the myocardium following UTMD. (A)** Immunohistochemistry results displayed that the expression of SDF-1 significantly increased in the UTMD group compared with the ultrasound, MB and control groups. Bar, 100 μm. **(B)** Protein expression of SDF-1 determined by Western blotting in ischemic myocardium from 4 groups with GAPDH as the internal control. **(C)** Expression of SDF-1 quantified by densitometric scanning was highest in the UTMD group. Values are mean ± SD. Significant differences were determined by using one-way ANOVA. N = 10/group. *p<0.0125 vs. Control; # p<0.0125 vs. MB; & p<0.0125 vs. Ultrasound. **(D)** Protein level of SDF-1 expression in myocardium quantified by ELISA, significantly increased in the MI+UTMD group. **(E)** Distribution of grafted BMSCs in the myocardium and other organs. Values are mean ± SD. Significant differences were determined by using one-way ANOVA. N = 10/group. *p<0.0125 vs. Sham; # p<0.0125 vs. MI; & p<0.0125 vs.MI+SDF-1.

After UTMD treatment, rats were sacrificed to examine the expression of SDF-1 by immunohistochemistry and Western blotting. Both analyses revealed that UTMD treatment markedly increased the expression of SDF-1 compared with the control, MB, and ultrasound groups (**Figures 6A-C**). Also, increased expression of SDF-1 in the infarction border area in MI, MI+SDF-1 and MI+UTMD groups at days 1, 3, and 4 peaking at day 3 was observed by ELISA. However, the SDF-1 protein level significantly boosted in the MI+UTMD group compared with MI and MI+SDF-1 groups (**Figure 6D**). As shown in **figure 6E**, the lungs had the highest percentage of BMSC homing compared with other organs in all groups, and UTMD significantly increased the homing of BMSCs to the infarcted myocardium compared with other groups.

### PHD2 silencing increases BMSC survival following transplantation by UTMD

**Figure 7:**
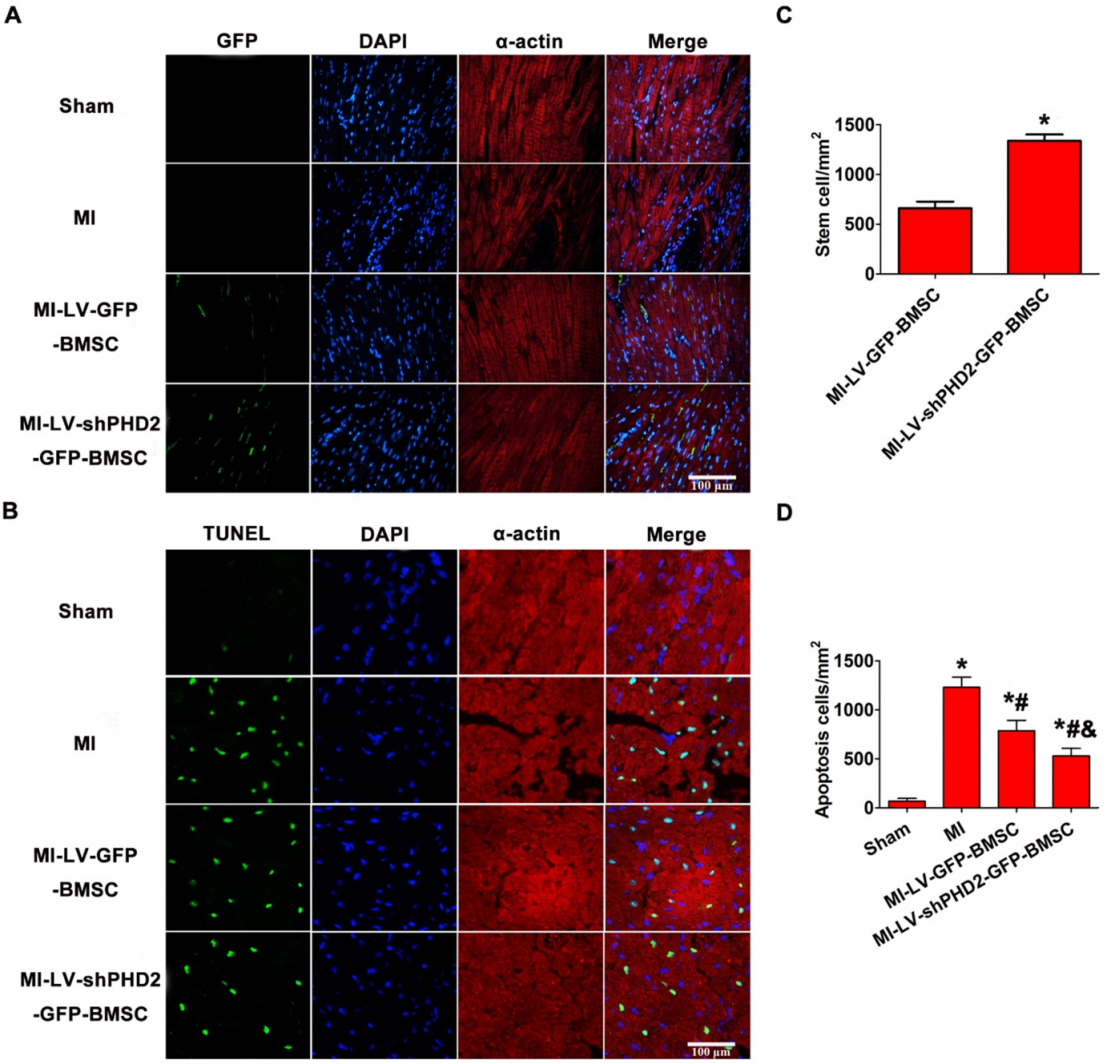
** PHD2-shRNA modification increased BMSC survival and reduced cardiomyocyte apoptosis in the infarct border area after transplantation by UTMD. (A)** There were no fluorescence cells in the infarct border area in the Sham and MI groups. Several EGFP-positive cells were observed in the MI-LV-GFP-BMSC group. In contrast, numerous EGFP-positive cells were observed in the MI-shPHD2-GFP-BMSC group. Cardiomyocytes were labeled by immunofluorescent histochemical staining with anti-α-actin antibody and cell nuclei were stained with DAPI. Bar, 100 μm. **(B)** Quantitative study of the EGFP-positive cells after post-MI transplantation. Values are mean ± SD. Significant statistic difference was determined by Student's t-test. N = 10/group. * P < 0.05 vs. MI-LV-GFP-BMSC. **(C)** TUNEL assay was applied to detect cardiomyocyte apoptosis in the Sham, MI, MI-LV-GFP-BMSC, and MI-LV-shPHD2-GFP-BMSC groups. Cardiomyocytes were labeled with anti-α-actin antibody and cell nuclei with DAPI. Bar, 50 μm. **(D)** Quantitative study of the apoptotic cell ratios by calculating the number of positive cells per square micrometer area in the gene transfected cardiomyocytes. Contrary to other treatment groups, apoptotic cardiomyocytes significantly decreased in the MI-LV-shPHD2-GFP-BMSC group in the infarct border zone at 48 hours post-MI. Values are mean ± SD. Significant differences were determined by one-way ANOVA. N = 10/group. * P < 0.0125 vs. Sham; # P < 0.0125 vs. MI; & P < 0.0125 vs. MI-LV-GFP-BMSC.

The transplanted GFP+ cells were detected by fluorescence microscopy in the peri-infarct myocardial tissues 48 h after BMSC transplantation. As shown in **Figure 7A-B**, no GFP+ cells were found in the sham and MI groups, and only a few GFP+ cells were observed in the MI-LV-GFP-BMSC group. However, a large number of GFP+ cells were noticed in the MI-LV-shPHD2-GFP-BMSC group, confirming that PHD2 silencing enhanced BMSC activity. BMSC transplantation into myocardial tissue with the use of the UTMD technique was, therefore, performed. PHD2-shRNA modification increased the activity of transplanted stem cells after they migrated into ischemic myocardial tissue following UTMD.

### Effects of PHD2 shRNA-modified BMSCs on post-MI myocardial remodeling

Myocardial cell apoptosis in the infarct border zone was measured by the TUNEL assay to determine the mechanism underlying the protective effects of PHD2 shRNA-modified BMSC transplantation. TUNEL+ cardiomyocyte nuclei were moderately decreased in MI-LV-GFP-BMSC-treated hearts but were significantly decreased in MI-LV-shPHD2-GFP-BMSC-treated hearts at 48 h post-MI (**Figure 7C-D**).

### Increased cardiac function and varied infarct morphology after transplantation of PHD2-shRNA-modified BMSCs

**Figure 8:**
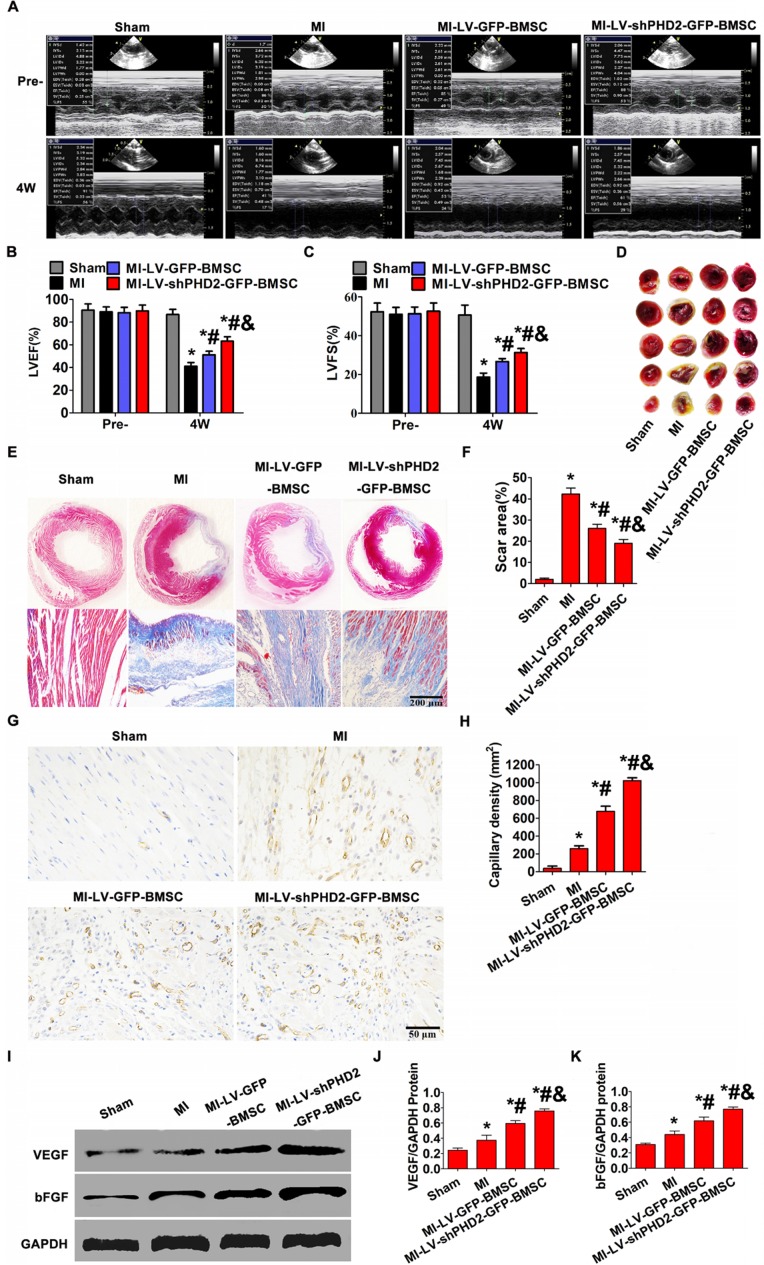
** Increased cardiac function and reduced infarct size via promoting angiogenesis after PHD2-shRNA-modified BMSC transplantation by UTMD. (A)** Representative M-mode images of hearts with sham surgery or MI in the 4th week after BMSC transplantation by UTMD. **(B-C)** Left ventricle ejection fraction (LVFS) and left ventricle fractional shortening (LVEF) measured at the 4th week were highest in the MI-LV-shPHD2-GFP-BMSC group. **(D)** TTC staining of myocardial segments in each group. The survived myocardium is stained red and the infarcted myocardium is stained white. **(E)** Representative Masson's trichrome-stained histological sections from Sham, MI, MI-LV-GFP-BMSC, or MI-LV-shPHD2-GFP-BMSC groups at the 4th week. Bar, 200 μm. **(F)** The infarct size, expressed as a percentage of the total tissue area, was noticeably down-regulated in the MI-LV-shPHD2-GFP-BMS group in contrast to MI-LV-GFP-BMSC group. **(G)** Representative photomicrographs showing capillary density in various experimental groups by CD31 immunostaining on the day 28th after BMSCs transplantation by UTMD. Bar,50 μm. **(H)** Quantitative study of the numbers of capillary vessels in different treatment groups. There were significantly more capillary vessels in the MI-LV-shPHD2-EGFP-BMSC group compared to other groups. **(I)** Protein expression of VEGF and bFGF determined by Western blotting in ischemic myocardium from four groups, with GAPDH as the internal control. **(J-K)** Quantitative study of the expression levels of VEGF and bFGF in various treatment groups. The expression levels of VEGF and bFGF were highest in the MI-LV-shPHD2-EGFP-BMSC group. Values are mean ± SD. Significant differences was determined by using Student's t-test. N =10/group. * P < 0.0125 vs. Sham; # P < 0.0125 vs. MI; & P < 0.0125 vs. MI-LV-GFP-BMSC.

To determine whether PHD2 silencing enhanced therapeutic effect of BMSCs, cardiac function and infarct size in post-MI rat hearts were evaluated following BMSC transplantation. Echocardiography was used to estimate cardiac function. LVFS and LVEF were similar among all groups before surgery. In the 4th week after BMSC transplantation by UTMD, the MI group showed severely impaired LV contractile function (LVFS and LVEF) compared with the sham group. Conversely, the highest LVEF and LVFS were observed in the MI-LV-shPHD2-GFP-BMSC group (**Figure 8A-C**), which were significantly greater than those in the MI-LV-GFP-BMSC group.

Histopathology examination of rat hearts was performed at the 4th week after BMSC transplantation by UTMD. In the MI group, considerable scar formation was observed, and the scar size decreased in the groups that received BMSC transplantation. As anticipated, the myocardial infarction size was smaller in the MI-LV-shPHD2-GFP-BMSC group than that in the MI-LV-GFP-BMSC group (**Figure 8D-F**). Thus, the UTMD-mediated localized myocardial delivery of PHD2 shRNA-modified BMSCs protected the heart from infarction and ameliorated cardiac function.

### PHD2 shRNA-modified BMSCs promote neovascularization *in vivo*

We next tested whether PHD2 shRNA-modified BMSCs could promote neovascularization *in vivo*. Immunohistochemical staining for CD31 was performed to assess the effect of PHD2 shRNA-modified BMSCs on revascularization in ischemic myocardium. Blood vessel density at the 4th week after transplantation was noticeably different between the MI-LV-shPHD2-GFP-BMSC group and the MI-LV-GFP-BMSC or MI groups in the peri-infarct regions. The highest number of blood vessels in the peri-infarct areas of the MI-LV-shPHD2-GFP-BMSC group was observed compared with MI-LV-shPHD2-BMSC and MI groups (**Figure 8G-H**). Furthermore, PHD2 shRNA-modified BMSC transplantation by UTMD promoted VEGF and bFGF expression in ischemic myocardium at the 4th week after AMI, which was significantly higher in MI-LV-shPHD2-GFP-BMSC group than in other groups (**Figure 8I-K**).

## Discussion

We employed a novel non-invasive application, targeted-gene-modified BMSC transplantation by UTMD, to deliver BMSCs with or without PHD2 shRNA modification into ischemic myocardia after AMI in rats. The underlying mechanism might be associated with the increased expression of SDF-1 in myocardial tissue following UTMD. MI-LV-GFP-BMSCs reduced cardiomyocyte apoptosis and infarct size, increased vascular density, and ameliorated cardiac function. However, MI-LV-shPHD2-GFP-BMSCs protected heart injury more effectively. Also, PHD2-shRNA modification induced higher BMSC survival both *in vitro* and *in vivo*, which was adversely affected by HIF-1α. Treatment with OGD increased cardiomyocyte apoptosis, which was significantly blunted by CM from shPHD2-GFP-BMSCs due to the increased secretion of IGF-1. Thus, UTMD combined with PHD2-shRNA modification could further enhance the effectiveness of stem cell therapy after AMI.

Ultrasound combined with microbubbles for stem cell therapy is an emerging technique 6, 20. In this study, UTMD was effectively used to promote BMSC delivery into ischemic myocardium. However, the mechanism of BMSC transplantation mediated by UTMD remains unclear. According to previous studies, UTMD produced pores in the capillary wall and widened the gaps between vascular endothelial cells, which led to an increase in permeability of myocardial blood capillary and could help promote the migration of transplanted BMSCs to the ischemic region 6, 21. Furthermore, the cavitation effect generated by UTMD induced a local inflammatory response and enhanced the adhesion of transplanted BMSCs to endothelial cells 21, 22. In this study, we found that UTMD significantly induced SDF-1 secretion and promoted BMSC transplantation in myocardial tissues (Figure 6), which was consistent with previous findings 7, 22. It has been reported that SDF-1α/CXCR4 axis plays a crucial role in stem cell mobilization, chemotaxis, homing, and engraftment in the repairment of infarcted myocardium 23, 24. SDF-1, released by cells within the impaired myocardium, is an essential chemokine inducing BMSC migration 25.

The cavitation effect generated by UTMD can facilitate transplanted BMSC migration to the ischemic region. However, microbubble destruction might also cause some tissue injury, including apoptosis and necrosis of cardiomyocytes 22. Different frequencies of the ultrasound lead to diverse degrees of tissue injury and generate varying local microenvironments 22. According to a previous study, ultrasound with a low intensity of 2 W/cm^2^ and a frequency of 1 MHz did not adversely affect cell viability 18; therefore, the identical ultrasonic parameters were adopted here. The results demonstrated that ultrasound (2 W/cm^2^, 1MHz), in combination with MBs, did not noticeably impact myocardial apoptosis *in vivo*.

BMSC transplantation has been a promising avenue for the treatment of damaged myocardium 26, 27. However, simple BMSC transplantation by UTMD has limitations because of the poor survival of transplanted stem cells. The primary factor leading to transplanted cell death is considered to be the limited blood supply in the infarct zone generating hypoxia and oxidative stress 28, 29. Accordingly, it is important to enhance the viability of stem cells through genetic modification prior to stem cell transplantation using UTMD technology. A better option might be to modify the stem cells with HIF-1α because it regulates >60 genes that control cell survival and metabolism in the poor conditions 30. Under normoxia, however, HIF-1α is hydroxylated by PHD2, ubiquitinated, and eventually degraded 13. Recently, RNA interference has emerged as a useful tool for gene silencing. Here, we selected PHD2 as the knockdown target and designed an shRNA against PHD2 for stabilizing endogenous levels of HIF-1α protein by inhibiting its degradation. To accomplish this, a lentiviral vector against PHD2 was successfully recombined and effectively transfected into BMSCs. The PHD2 expression was much lower and the HIF-1α expression was much higher in the BMSCs modified with PHD2 shRNA than in BMSCs or BMSCs modified with GFP (Figure 2). Furthermore, PHD2 silencing in BMSCs improved BMSC survival (Figure 3), reduced cardiomyocyte apoptosis (Figures 4 and 7), decreased infarction size, increased micro- vessel density, and then improved cardiac function in MI rats (Figure 8).

We proceeded to evaluate the mechanism involved in PHD2 shRNA-mediated cell protection. As shown in Figures 3 and 7, PHD2 silencing significantly increased HIF-1α abundance, thus increasing BMSC survival *in vivo* and *in vitro*. However, the cytoprotective effects of PHD2 silencing on BMSCs were entirely abolished, when HIF-1α was silenced in BMSCs, which suggested that PHD2 silencing decreased BMSC death by a HIF-1α-dependent pathway (Figure 3). Besides its role in BMSC survival, PHD2 silencing in BMSCs could incur the myocardial effects by inhibiting cardiomyocyte apoptosis both *in vitro* (Figure 4) and *in vivo* (Figure 7). During *in vitro* studies, the CM from PHD2 shRNA-modified BMSCs exhibited a cytoprotective effect by inhibiting cell apoptosis of cardiomyocytes when exposed to OGD. On the contrary, cardiomyocytes cultured in CM from BMSCs modified with GFP or nonconditioned CM did not display the same cytoprotective effect (Figure 4). PHD2 shRNA-modified BMSC transplantation mediated by UTMD also significantly reduced myocardial apoptosis *in vivo* (Figure 7). It has previously been shown that PHD2 silencing in transplanted cells decreased cardiomyocyte apoptosis via a protective paracrine mechanism mediated by facilitating IGF-1 secretion followed by NF-κB signaling pathway activation 31. Here, IGF-1, a protective cytokine for ischemic cardiomyocytes, was shown to be noticeably upregulated *in vitro* after PHD2 silencing (Figure 4).

As demonstrated by previous studies, stem cells rarely differentiated into cardiomyocytes 31, 32, and the paracrine function was considered as the principal mechanism for the conducive influence exerted by transplanted BMSCs. In addition to the secretion of IGF-1, a protective cytokine for ischemic cardiomyocytes, our results also revealed that PHD2 silencing promoted the secretion of vascular growth factors, including VEGF and bFGF, which promoted new blood vessel network formation and adjusted blood supply of cardiac muscle (Figure 8). PHD2 gene silencing has been shown to stimulate the hypoxia response pathway mediated by HIF-1α under the anoxic condition and to facilitate the secretion of vascular growth factors 14. As displayed in Figure 8, the cell-based delivery of PHD2 shRNA ameliorated LV contractile function, in addition to attenuation of cardiac remodeling, e.g., LV wall thinning and reducing infarcted scar size. The implication of PHD2 silencing in such a recovery process for the post-MI heart may be realized from the subsequent paracrine function, which enhanced cell survival through the release of growth factors.

In conclusion, the present study demonstrated that UTMD combined with PHD2 shRNA modification increased the therapeutic effect of grafted cells via improving BMSC survival, reducing cardiomyocyte apoptosis, decreasing fibrosis, and increasing microvessel density in the target area. The combination of UTMD and PHD2 shRNA modification represents an emerging potential approach for cellular therapy to treat AMI.

## Figures and Tables

**Table 1:** Primers used for RT-PCR

Genes	Primer	Sequence	Product size
PHD2	Sense	5'-TACAGGATAAACGGCCGAAC-3'	209bp
	Antisense	5'-TTGGGTTCAATGTCAGCAAA-3'	
HIF-1α	Sense	5'-CGCAGTGTGGCTAC AAGAAA-3'	205bp
	Antisense	5'-TAAAT TGAACGGCCCAAAAG-3'	
SRY	Sense	5'-CATCGAAGGGTTAAAGTGCCA-3'	459bp
	Antisense	5'-ATAGTGTGTAGGTTGTTGTCC-3'	
β-actin	Sense	5'-TGACGTGGACATCCGCAAAG-3',	240dp
	Antisense	5'-CTGGAAGGTGGACAGCGAGG-3'	

**Table 2:** Primary antibodies used for Western blots and immunohistochemistry

Antibody	Dilution	Source	Catalog #
HIF-1α	1:500	Bioworld	BS3514
PHD2	1:100	Santa Cruz	sc-271835
EPO	1:500	Abcam	sab226956
Activated caspase-3	1:500	Abcam	ab49822
Total caspase-3	1:100	Abcam	ab4051
SDF-1	1:500	Abcam	ab25117
α-actin	1:100	Abcam	ab137346
CD31	1:50	Santa Cruz	sc-71873

**Table 3:** Microbubble (MB) characterization

	Concentration (×10^9^/ml)	zeta potential(mV)	Average diameter (μm)
**MB**	4.12 ± 0.29	27.18 ± 3.32	1.07 ± 0.21
