# Myocardium-targeted transplantation of PHD2 shRNA-modified bone mesenchymal stem cells through ultrasound-targeted microbubble destruction protects the heart from acute myocardial infarction: Erratum

**DOI:** 10.7150/thno.72333

**Published:** 2022-03-06

**Authors:** Zhenxing Sun, Yuji Xie, Robert J. Lee, Yihan Chen, Qiaofeng Jin, Qing Lv, Jing Wang, Yali Yang, Yuman Li, Yu Cai, Rui Wang, Zhengyang Han, Li Zhang, Mingxing Xie

**Affiliations:** 1 Department of Ultrasound, Union Hospital, Tongji Medical College, Huazhong University of Science and Technology, Wuhan 430022, China.; 2 Hubei Province Key Laboratory of Molecular Imaging, Wuhan 430022, China; 3 College of Pharmacy, The Ohio State University, Columbus, OH 43210, USA

The authors apologize that the original version of this paper unfortunately contained two incorrect figures and a terminology error due to negligence in drafting and proofreading. Firstly, in Section A of Figure 2, the DAPI and Merge images of the control group were not presented correctly. Secondly, Section B and Section C of Figure 7 were reversely marked. In addition, the term MI-LV-shPHD2-BMSC in the last paragraph of the Results section should be “MI-LV-GFP-BMSC” (Page 4977).

The authors apologize for any inconvenience these errors may have caused. Luckily they have no influence on the conclusion drawn by the study. The correct figures are shown below.

## Figures and Tables

**Figure 1:**
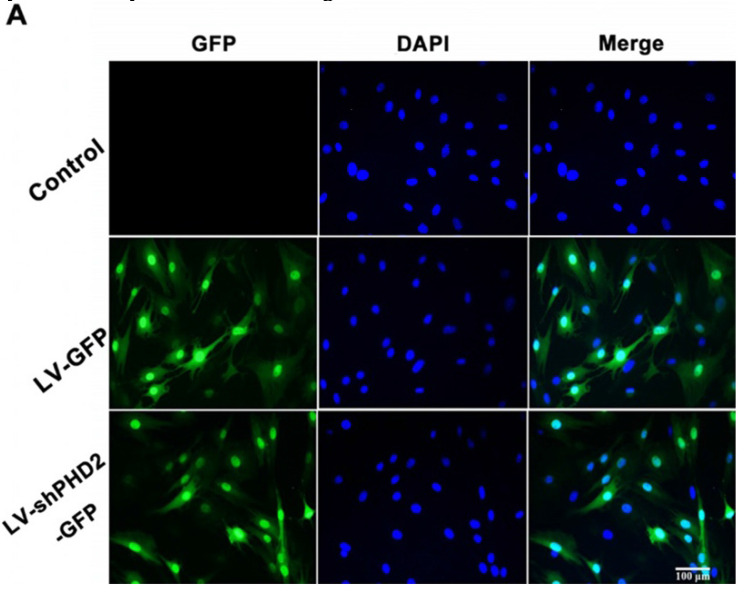
Corrected figure for original Figure 2A.

**Figure 2:**
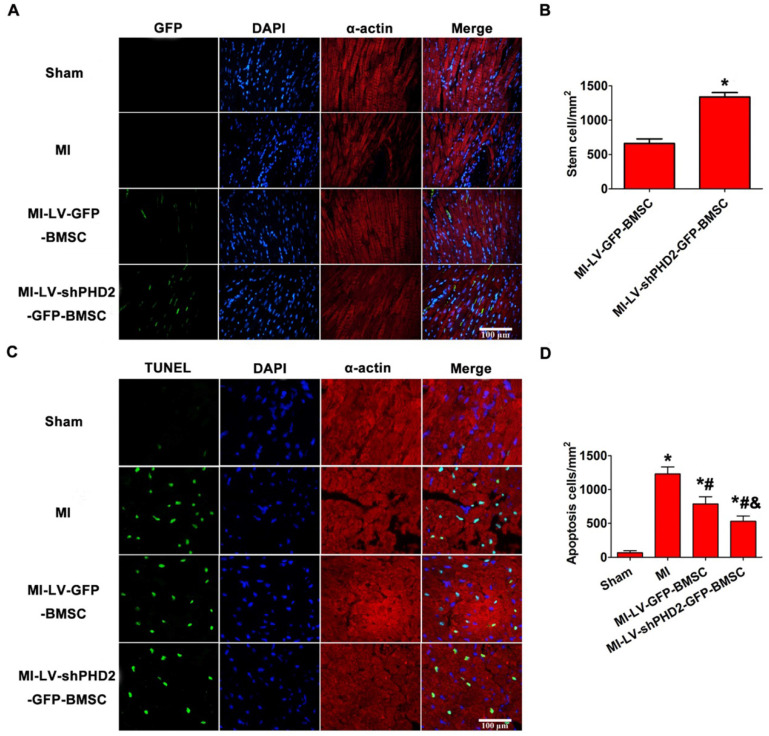
Corrected figure for original Figure 7.
