# Supplementary material for: Myocardium-targeted transplantation of PHD2 shRNA-modified bone mesenchymal stem cells through ultrasound-targeted microbubble destruction protects the heart from acute myocardial infarction
Source: Theranostics. 2020 Apr 6;10(11):4967–82. doi: 10.7150/thno.43233 (PMC7163444; doi:10.7150/thno.43233)
Supplement: Supplementary file 1 — Supplementary figures and tables. [file thnov10p4967s1.pdf]

Myocardium-targeted transplantation of PHD2 shRNA-modified bone mesenchymal stem cells through ultrasound-targeted microbubble destruction protects the heart from acute myocardial infarction

Zhenxing Sun [1,2*], Yuji Xie [1,2*], Robert J. Lee [3], Yihan Chen [1,2], Qiaofeng Jin[1,2], Qing Lv [1,2], Jing Wang [1,2], Yali Yang [1,2], Yuman, Li [1,2], Yu Cai [1,2], Rui Wang [1,2], Zhengyang Han [1,2], Li Zhang [1,2☒], Mingxing Xie [1,2☒]

[1]Department of Ultrasound, Union Hospital, Tongji Medical College, Huazhong University of Science and Technology, Wuhan 430022, China.

[2]Hubei Province Key Laboratory of Molecular Imaging, Wuhan 430022, China

[3]College of Pharmacy, The Ohio State University, Columbus, OH 43210, USA

Category of the Manuscript: Original Article

Total Word Count of the Manuscript:8029

[*]These authors contributed equally to this article.

[☒]**Co-Corresponding author**:

1. Mingxing Xie, MD, Ph.D.; 1277 Jiefang Avenue, Wuhan, China. Tel: 86-27-85726430; Fax: 86-27-85726386. E-mail address: xiemx@hust.edu.cn.
2. Li Zhang, MD, Ph.D.; 1277 Jiefang Avenue, Wuhan, China. Tel: 86-27-85726430; Fax: 86-27-85726386. E-mail address: zli429@hust.edu.cn.

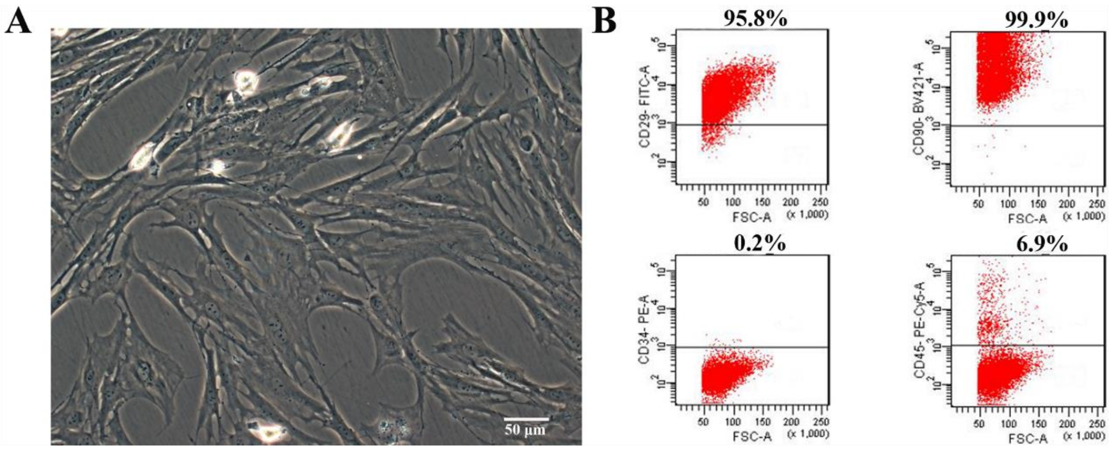

Figure S1. Characterization of BMSCs by microscopy and by flow cytometry.

A. The morphology observation of BMSCs under the optical microscopy. Bar, 50μm; B. Flow cytometry of BMSCs for mesenchymal CD29, 90, 34, and 45 markers. Unstained cells acted as control.
